# Revealing the anticancer potential of nano-encapsulated graviola extract on tongue carcinoma (SCC154) cell line: targeting the PI3K/AKT/mTOR pathway (in vitro study)

**DOI:** 10.1186/s12906-025-05113-4

**Published:** 2025-10-02

**Authors:** Amany Hany Mohamed Kamel, Ahmed A. Abd-Rabou, Ahmed Basuoni, Nermeen AbuBakr

**Affiliations:** 1 Oral Biology Department, Faculty of Dentistry, Cairo University, Cairo, Egypt; 2 Hormones Department, Medical Research and Clinical Studies Institute, National Research Centre, Dokki, Giza, Egypt; 3 Faculty of Medicine, Cairo University, Cairo, Egypt

**Keywords:** Graviola, Antineoplastic agents, Nanoparticles, Carcinoma, Tongue

## Abstract

**Background:**

Graviola emerged as a promising anticancer agent, with nanotechnology enhancing drugs’ therapeutic potential. The purpose of this work was to explore graviola extract and its nano-platform’s effects on tongue carcinoma (SCC154 cells) in vitro model.

**Methods:**

Graviola leaves extract (GLE) was isolated, and its phenolic content was identified. Three nano-formulations (F1-F3) were optimized for GLE delivery, with F1 chosen for its optimal size and stability to synthesize graviola nanoparticles (GNPs). SCC154 cells were split into three groups: group Ι (untreated SCC154 cells), group ΙΙ: SCC154 cells + ethanolic GLE, and group ΙΙΙ: SCC154 cells + GLE encapsulated in a nano-void delivery system (GNPs). In vitro tests assessed cell viability via MTT assay, cell cycle, and apoptosis by flow cytometry, DNA damage using comet assay, and gene expression of the key molecular markers (PI3K, AKT, mTOR, and GSK-3β) by quantitative real-time polymerase chain reaction. Transmission electron microscopic examination of cells was also performed.

**Results:**

GLE and GNPs reduced SCC154 cells’ proliferation compared to untreated cells, with GNPs showing significantly higher cytotoxicity. Both treatments also induced apoptosis, arrested the cell cycle, and caused DNA damage with a significant pronounced effect in the GNPs-treated group. Gene expression analysis revealed a substantial decline in PI3K, AKT, mTOR, and GSK-3β in both treated groups relative to the control group, with a significant downregulation in the GNPs-treated group. Ultrastructural examination revealed severe destruction in tongue carcinoma cells of both treated groups, with substantial damage in the GNPs-treated group.

**Conclusion:**

GNPs showed a better impact than GLE in tongue carcinoma therapy, causing cytotoxicity and apoptosis, potentially through the PI3K/AKT/mTOR pathway.

**Supplementary Information:**

The online version contains supplementary material available at 10.1186/s12906-025-05113-4.

## Introduction

Cancer still poses a serious threat to global health, with standard therapies relying on cytotoxic agents, known for their detrimental effects on healthy cells [1]. Thus, there is a pressing need to explore alternative cancer treatment paradigms that selectively target cancer cells while minimizing toxicity to normal cells [2].

Among the key signaling pathways in cancer, the phosphatidylinositol-3-kinase (PI3K)/protein kinase B (AKT)/mammalian target of rapamycin (mTOR) cascade plays a pivotal role in regulating cellular survival, and apoptosis. Dysregulation of this pathway is a hallmark of many tumors, making it an appealing target for multiple therapies [3].

Natural products, rich in various phytochemicals, have shown significant efficacy against cancer [2, 4]. The Annonaceae family includes the herb graviola (Annona muricata), which is recognized as a natural source of over a hundred acetogenins [5]. Graviola’s effectiveness was proven in restraining cancerous cells across various human cell lines while maintaining a benign impact on normal cells [6]. Nanoparticles (NPs) enable safe administration of such bioactive compounds at effective doses [7]. These nano-carriers enhance the bioavailability of therapeutics, allowing targeted delivery to tumor sites while minimizing systemic toxicity [8–10].

Despite the reported anticancer potential of graviola on numerous cancer cell lines, there are very few studies focusing specifically on oral squamous cell carcinoma. To the best of our knowledge, the current investigation is the first to assess nano-encapsulated graviola against oral carcinoma cells via PI3K/AKT/mTOR pathway targeting. Using SCC154 cells, we compared the anticancer effects of graviola extract in both its conventional and nano-formulated forms.

## Materials and methods

### Preparation of graviola extract

After soaking the powdered graviola leaves (supplied by NOW Foods, USA) in ethanol at room temperature, the mixture was extracted for 48 h, with intermittent shaking. After filtering, the extract was evaporated at 60 °C under reduced pressure until completely dry. The dry extract was then sealed in a container and designated as graviola leaves extract (GLE) [11].

### Analysis of total phenolic content and specific phenolic compounds

20 µl of GLE and 100 µl of Folin-Ciocalteu reagent were combined in a flask. To neutralize the solution, 80 µl of saturated sodium carbonate was added after the mixture had been incubated for 15 min. The mixture’s absorbance at 760 nm was measured after two further hours of incubation using a UV-visible spectrophotometer [12]. A typical calibration curve for gallic acid was used to calculate the total phenolic content. High-performance liquid chromatography (HPLC) was used to further evaluate the phenolic components [7, 11]. Phenolic compounds were separated using a Hewlett Packard Series 1050 HPLC system (USA), equipped with a C18 Hypersil BDS column. Detection of phenolic compounds was performed using a UV detector set at 280 nm.

### Optimization and encapsulation of nano-formulations for GLE delivery

Three distinct nano-formulations (F1-F3) were developed as potential carriers. For F1, a mixture containing 1.5 mMol of 1-ethyl-3-(3-dimethylaminopropyl)-N’-ethyl-carbodiimide (EDC) and 1.6 mMol of N-hydroxysuccinimide (NHS) was prepared. Heparin (0.1 mMol) was then coupled with 0.1 mM of polyethylene glycol amine (PEG-NH2, MW 5000 Da) and 0.1 mM of poly (D, L-lactide-co-glycolide) (PLGA, MW 7000 Da) at a ratio of 1 PEG: 1 PLGA using the EDC/NHS mixture. The resulting mixture underwent sonication for 5 min and stirring for 24 h. For F2 and F3, similar procedures were followed, with varying ratios of PEG-NH2 to PLGA (2:1 for F2 and 3:1 for F3).

### Characterization of graviola nanoparticles

#### Dialysis and measurement of entrapment efficiency

Graviola nanoparticles (GNPs) were subjected to dialysis for 3 h using a membrane bag (Spectra/Por membrane; molecular weight cut-off, MWCO: 25,000 Da) against distilled water. The amount of GLE retained within the NPs was quantified spectrophotometrically based on a standard calibration curve. Entrapment efficiency % (EE%) was calculated using the following formula: EE% = (Total drug − Free drug​)/Total drug ×100.

#### Particle size, zeta potential and polydispersity index analysis

Laser diffractometry and photon correlation spectroscopy were used to analyze the particle size of the GNPs. One milliliter of the nanoparticle solution was put into a disposable transparent sized clear cuvette to perform measurements. A Malvern Zetasizer was used to measure the nano-sample’s size, zeta potential, and polydispersity index (PDI) at 25 °C.

#### In vitro release analysis

The release profile of GLE and GNPs was analyzed using the dialysis bag diffusion method. A known amount of GLE or GNPs, equivalent to an equal amount of graviola, was put in pre-soaked dialysis membranes (MWCO: 12 kDa) and immersed in 100 mL of phosphate-buffered saline (PBS) at 37 ± 0.5 °C with constant stirring at 100 rpm. At certain time intervals (0 to 24 h, every 2 h), 5 mL of the external medium was withdrawn and replaced with fresh PBS. Drug release was quantified by measuring absorbance at 270 nm using a UV-visible spectrophotometer.

#### Transmission electron microscopic (TEM) analysis of GNPs

TEM (Philips CM-10, FEI Inc., Hillsboro, USA) was used to determine the shape of the GNPs. The nano-suspension was applied to copper grids coated with formvar at a dosage of 100 µg/mL. Once the samples were totally dry, they were dyed using a 2% w/v uranyl acetate solution. Images were taken and analyzed utilizing digital microscopy and soft imaging viewer software.

### In vitro experiments

#### Cell culture

Dulbecco’s Modified Eagle’s Medium, which contains 4.5 g/L glucose, L-glutamine, 10% fetal bovine serum, and antibiotics (penicillin–streptomycin), was utilized to cultivate human tongue carcinoma SCC154 cells (ATCC, USA). For the best cellular growth and proliferation, the cultures were kept at 37 °C with 5% CO_2_ in a humidified incubator.

### Dosage of graviola and nano-graviola extracts

The exact concentration used for graviola extract was 51.4 µg/mL, and for nano-graviola extract was 27.3 µg/mL. This was based on an initial test conducted, where the half maximal inhibitory concentration (IC50) was determined by measuring cell viability using the MTT test.

### Experimental design

Three groups of cultured human tongue cancer cells were created: group I: SCC154 cells, serving as the untreated control group; group II (GLE): SCC154 cells treated with ethanolic GLE; and group III (GNPs): SCC154 cells treated with GLE encapsulated in a nano-void delivery system. All cultured carcinoma cells in groups (Ι, ΙΙ, and ΙΙΙ) were subjected to:

### Cell viability assessment (MTT assay)

SCC154 cells were planted at a density of 1 × 10⁴ cells/well in 96-well plates. After a day, the cell culture media was supplemented with the IC50 of GLE and its NPs. Following another 24-hour incubation, MTT solution (5 mg/mL in PBS) was applied to each well, and the plates were subsequently incubated for a further 4 h at 37 °C. Dimethyl sulfoxide was utilized to dissolve the resulting dark blue, water-insoluble formazan crystals. Absorbance was determined at 450 nm using a microplate reader. The percentage of cell viability was computed in relation to the control group [13].

### Apoptosis assessment (flow cytometry)

A count of 1 × 10^6^ cells was used for seeding, and the cells were left to incubate for a full day. After treating the cells with the recommended therapies at their IC50 concentrations, they were cultured for an additional twenty-four hours. Following this treatment, annexin V FITC and propidium iodide (PI) were utilized to label the cells. The degree of apoptosis in treated and untreated cells was then measured using a flow cytometer (Beckman Coulter, USA). This process enabled the identification of necrotic populations, as well as the distributions of late and early apoptotic cells.

### Analysis of cell cycle (flow cytometry)

Measurements were taken to assess the distribution of cancer cells in the G0/G1, S, and G2/M phases of the cell cycle. The cells were cultured at a count of 2 × 10^5^ cells/mL and then subjected to the IC50 dosages of the suggested therapies. Trypsin was introduced to the cells after a 24-hour incubation period, and they were then centrifuged for five minutes at 1000 × g. A flow cytometer was used to examine the isolated cells after they had been treated with PI in accordance with the manufacturer’s instructions.

### DNA damage assessment (comet assay)

Alkaline conditions were used for the comet assay. A 0.5% normal agarose coating was applied to standard microscope slides. Cells were washed and mixed with 0.5% low-melting-point agarose and applied to the slides. The slides were immersed in a lysing solution at 4 °C for one hour. After lysis, slides were subjected to electrophoresis in an alkaline buffer for 20 min. After electrophoresis, slides were washed and neutralized with buffers containing 10 mM Tris. Ethanol was used for fixation, and DNA was stained with ethidium bromide. Fifty randomly chosen cells from each sample were measured using a comet assay automatic image analysis system with a Leica fluorescence microscope [14].

### Quantitative real-time polymerase chain reaction (qRT-PCR) analysis

The RNeasy Micro Kit (Qiagen) was used to isolate the total RNA. A high-capacity cDNA reverse transcription kit (Applied Biosystems, USA) was utilized to create cDNA from 1 µg of the extracted total RNA. An Applied Biosystems SYBR^®^ Green Master Mix kit was utilized for PCR analysis. The thermal profile was as follows: reverse transcription took 10 min at 45 °C, reverse transcriptase was inactivated for 2 min at 98 °C, and initial denaturation with 40 cycles of 10 s at 98 °C, 10 s at 55 °C, and 30 s at 72 °C for amplification. GAPDH served as an internal control [15]. Table 1 lists the primer sequences of the examined genes.

**Table 1:** Primers’ sequence of studied genes

Gene	Forward	Reverse
PI3K	5’-GGTTGTCTGTCAATCGGTGACTGT-3’	5’-GAACTGCAGTGCACCTTTCAAGC-3’
AKT	5’-TTCTGCAGCTATGCGCAATGTG-3’	5’-TGGCCAGCATACCATAGTGAGGTT-3’
mTOR	5’-GCTTGATTTGGTTCCCAGGACAGT-3’	5’ GTGCTGAGTTTGCTGTACCCATGT-3’
GSK-3β	5’-ACAGCAGCGTCAGATGCTAA-3’	5’-GGGACTGTTCAGGTGGAG-3’
GAPDH	5’-TGCACCACCAACTGCTTAGC-3’	5’-GGCATGGACTGTGGTCATGAG-3’

### TEM analysis of SCC154 cells

The cell suspension was fixed in 0.1 M phosphate buffer (pH 7.4) with 1% glutaraldehyde and 4% formaldehyde. One hour of post fixation was conducted using 1% osmium tetroxide in 0.1 M phosphate buffer. After being completely dehydrated, the cells were placed inside beam capsules and roasted for two days at 60 °C. Ultra-thin sections were cut out of the blocks. A Philips XL 30 transmission electron microscope was used to view these sections after they had been doubly stained with lead citrate and uranyl acetate [16].

### Statistical analysis

SPSS 18.0 was utilized to conduct the statistical analysis. The Kolmogorov-Smirnov analysis was utilized to verify the data for normalcy. The data was expressed using the mean ± standard deviation (SD). After comparing the groups using a one-way ANOVA, pairwise comparisons were conducted using Tukey’s post hoc test. A p-value of less than 0.05 was regarded as statistically significant.

## Results

### Identification of the ethanolic extract of graviola

The graviola extract with ethanol solvent exhibited very high phenolic content. There were six phenolic compounds identified using HPLC. Oleuropein, catechin, pyrogallol, gallic acid, caffeic acid, and ellagic acid were present in the GLE at different concentrations. Oleuropein and catechin were the major phenolics identified (Table 2).

**Table 2:** Quantification of total phenolic content and some phenolic compounds in GLE

Total phenolic content	250.12 ± 3.12 (mg GAE/g)
Compounds	Concentration (mg/g)
Oleuropein	69.25 ± 3.05
Catechin	52.46 ± 1.03
Pyrogallol	10.03 ± 0.50
Gallic acid	7.33 ± 0.36
Caffeic acid	2.73 ± 0.08
Ellagic acid	1.66 ± 0.06

### Characterization of the nano-formulations and selection of the optimal nano system for GLE delivery

The first nano-void formulation (F1), characterized by a 1:1 ratio of PLGA to PEG, exhibited the best size distribution, PDI, and zeta potential with a negative charge indicating a high level of stability followed by the second formulation (F2) with a 1:2 ratio of PLGA to PEG then the third formulation (F3) using a 1:3 ratio of PLGA to PEG. Therefore, F1 was used to encapsulate the GLE (Table 3).

### Physicochemical characterization of GNPs

The optimized GNPs showed a mean particle size of 140.7 ± 2.5 nm, a low PDI of 0.05 ± 0.02, and a zeta potential of −23.4 ± 3.5 mV, indicating a uniform, stable nano-system. Moreover, GNPs showed high entrapment efficiency of 92.7 ± 7.26%, indicating efficient encapsulation of the active compounds within the nanocarrier (Table 3).

**Table 3:** Average size, polydispersity index (PDI), zeta potential (ZP), and entrapment efficiency (EE %) of the synthesized NPs

NPs type	Ratio		Mean ± SD			EE %
	PLGA	PEG	Size, nm	PDI	ZP, mV	
Nano-void (F1)	1	1	120.4 ± 5.2	0.04 ± 0.01	−21.5 ± 4.7	-
Nano-void (F2)	1	2	136.5 ± 9.4	1.2 ± 0.50	−3.31 ± 1.5	-
Nano-void (F3)	1	3	189.13 ± 11.12	0.9 ± 0.36	+ 8.5 ± 2.26	-
GNPs	1	1	140.7 ± 2.5	0.05 ± 0.02	−23.4 ± 3.5	92.7 ± 7.26

Data were represented in terms of mean ± SD. Nano-void: nanoparticles without drug; F1, F2, F3, three different formulas

### In vitro release results

**Fig. 1:**
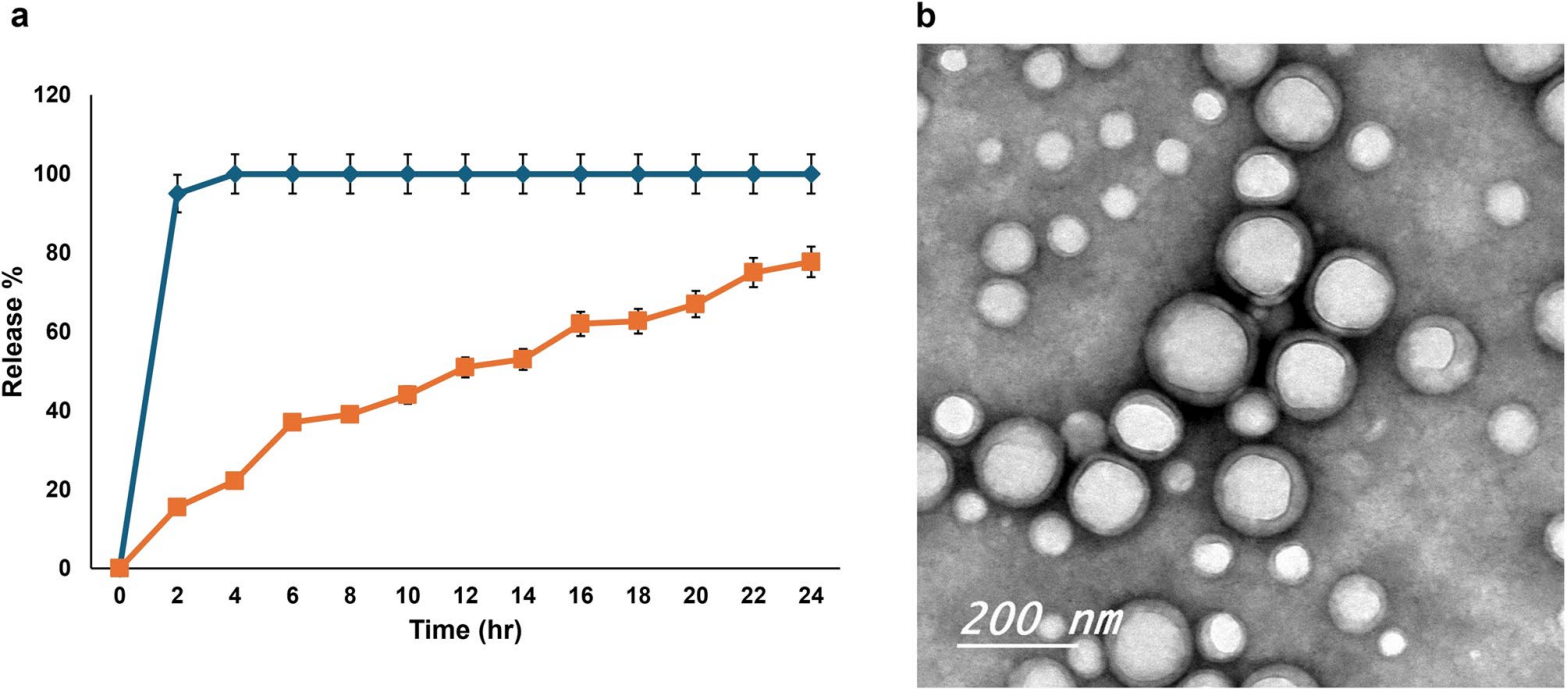
**a** In vitro release profile of GLE and GNPs over 24 h. **b** TEM image of GNPs showing GLE encapsulated within the core of NPs, surrounded by rounded membranes of PLGA and PEG (scale bar =200nm)

The in vitro release study revealed a substantial variation in the release behavior of free GLE compared to GNPs. GLE showed a rapid release pattern, with 95% ± 1.0% of the extract diffused within 2 h and complete release (100% ± 0.0%) by 4 h, indicating immediate diffusion of free graviola molecules. In contrast, GNPs exhibited a sustained release profile, with only 15.5% ± 3.4% released at 2 h, 22.2% ± 5.6% at 4 h, and 37.0% ± 5.6% at 6 h. By 12 h, 51.0% ± 4.9% was released, gradually increasing to 67.0% ± 7.3% at 20 h and reaching a maximum of 77.7% ± 5.8% at 24 h (Fig. 1a).

### TEM results of GNPs

The NPs exhibited a core-shell structure composed of a core surrounded by rounded membranes made of PLGA and PEG. The GLE was encapsulated within the core of the NPs (Fig. 1b).

### MTT assay results

**Fig. 2:**
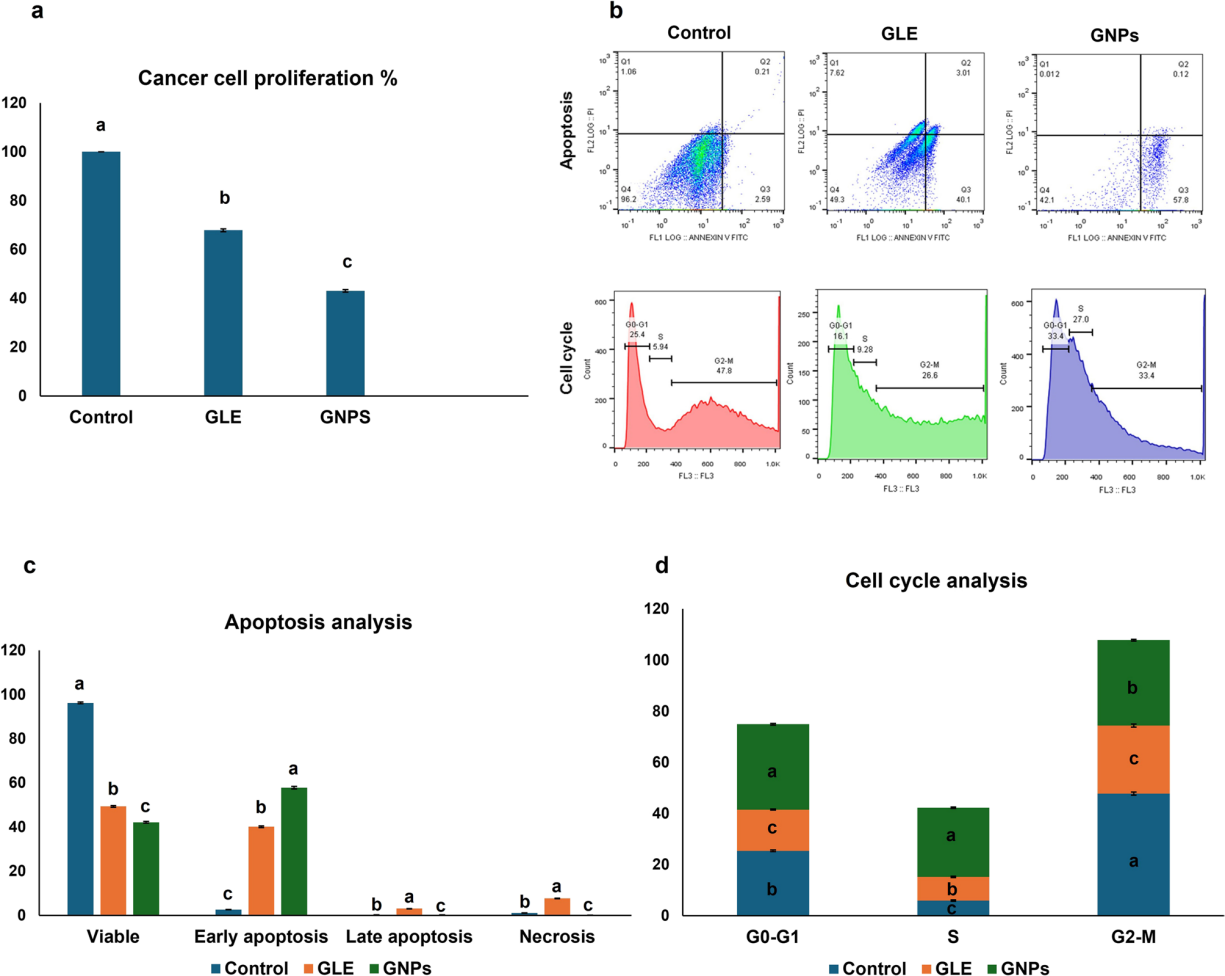
**a** Graph illustrating the mean ± SD of cancer cell proliferation (%) in each group measured by MTT assay. **b** Representative flow cytometry scatter plot (apoptosis assay) [Q1: necrotic cells (Annexin V⁻/PI⁺), Q2: late apoptotic cells (Annexin V⁺/PI⁺), Q3: early apoptotic cells (Annexin V⁺/PI⁻), Q4: viable cells (Annexin V⁻/PI⁻)] and histogram (cell cycle analysis). **c** Graph illustrating the mean ± SD of the percentages of viable, early apoptotic, late apoptotic, and necrotic cells in each group measured by apoptosis assay. **d** Graph illustrating the mean ± SD of the percentages of cells in each cell cycle phase in each group measured by cell cycle analysis. Significant differences exist between bars with various letters. Significance at *p*<0.05

There was a statistical difference in cell proliferation percentage among all groups using ANOVA (*p* < 0.001). Post-hoc pairwise comparisons showed a significant decline in tongue carcinoma SCC154 cells’ proliferation in GLE and GNPs groups compared to the untreated control group (*p* < 0.001). Furthermore, the GNPs group exhibited a significant decline in proliferation percentage compared to the GLE group (*p* < 0.001) (Fig. 2a).

### Apoptosis results

There was a statistical difference in viability, early apoptosis, late apoptosis, and necrosis among all groups using ANOVA (*p* < 0.001). Using Tukey’s post-hoc test, both GLE and GNPs groups revealed a significant decline in the percentage of viable cells compared to the untreated group, which showed the highest population of healthy cells (*p* < 0.001). Moreover, the graviola nano-regimen exhibited a significant decrease in viability compared to the GLE group (*p* < 0.001). In terms of early apoptosis, both GLE and GNPs significantly increased the percentage of early apoptotic cells relative to the control (*p* < 0.001), with GNPs inducing a significantly higher early apoptotic response than GLE (*p* < 0.001). For late apoptosis and necrosis, GLE showed the highest percentages of cells, followed by the control group, while the GNPs group exhibited the lowest values. These findings indicate that GNPs induce a more rapid and efficient apoptotic response, predominantly at the early stage (*p* < 0.05) (Fig. 2b and c).

### Cell cycle analysis results

The cell cycle was assessed by categorizing cells into three phases: the G0/G1 phase, DNA synthesis S phase, and the G2/M phase. There was a significant variation in each phase among all groups using ANOVA (*p* < 0.001). Post-hoc pairwise comparisons showed that in the G0/G1 phase, GLE significantly decreased the percentage of SCC154 cells compared to the control (*p* < 0.001), while GNPs caused a significant accumulation of cells at this phase (*p* < 0.001), indicating G0/G1 cell cycle arrest. In the S phase, both GLE and GNPs significantly elevated the percentage of cells compared to the control (*p* < 0.001), with GNPs showing an even more substantial increase in population of cells compared to GLE (*p* < 0.001), suggesting cell cycle arrest at this phase. In the G2/M phase, both treatments significantly reduced the cell population compared to the control (*p* < 0.001), with a further significant decline observed in the GLE group compared to the GNPs group (*p* < 0.001). These results suggest that GLE and GNPs affect cell cycle progression, with GNPs exerting a stronger arrest effect, primarily at G0/G1 and S phases (Fig. 2b and d).

### Comet assay results

**Fig. 3:**
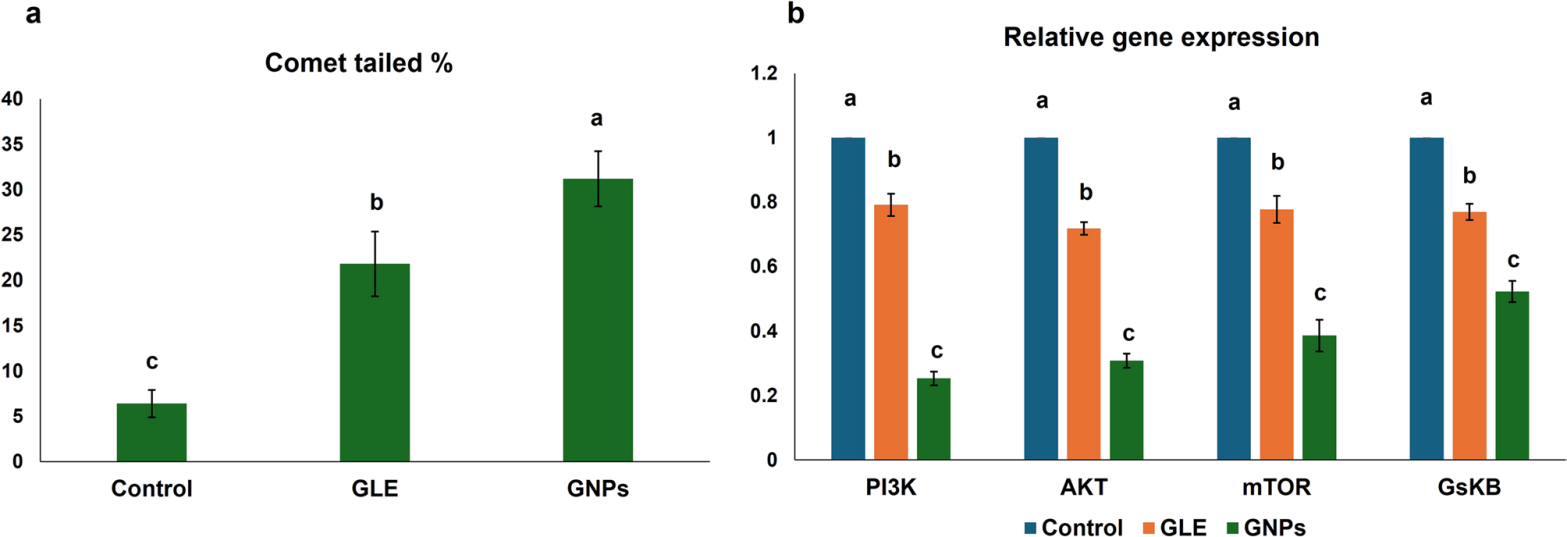
**a** Graph illustrating the mean ± SD of tailed DNA (%) in each group using comet assay. Significant differences exist between bars with various letters. Significance at *p*<0.05. **b** Graph illustrating the mean ± SD of mRNA expression levels of PI3K, AKT, mTOR, and GSK-3β in each group. Bars with various letters within each marker are significantly different. Significance at *p*<0.05

The assessment of comet assay data indicated a significant variation among all groups using ANOVA (*p* < 0.001). Using Tukey’s post-hoc test, both GLE and GNPs groups demonstrated significant elevation in tailed DNA % compared to the untreated group (*p* < 0.001). Additionally, the graviola nano-regimen exhibited an elevated percentage of tailed DNA compared to graviola extract (*p* < 0.001) (Fig. 3a).

### PCR results

The mRNA gene expression of all studied markers revealed a significant variation among all groups using ANOVA (*p* < 0.001). Post-hoc pairwise comparisons showed a significant downregulation in the mRNA levels of all markers in GLE, and GNPs groups compared to the untreated group (*p* < 0.001). The gene expression levels in cells treated with nano-graviola revealed an even more substantial downregulation in all studied genes compared to the GLE group (*p* < 0.001) (Fig. 3b).

### TEM results

**Fig. 4:**
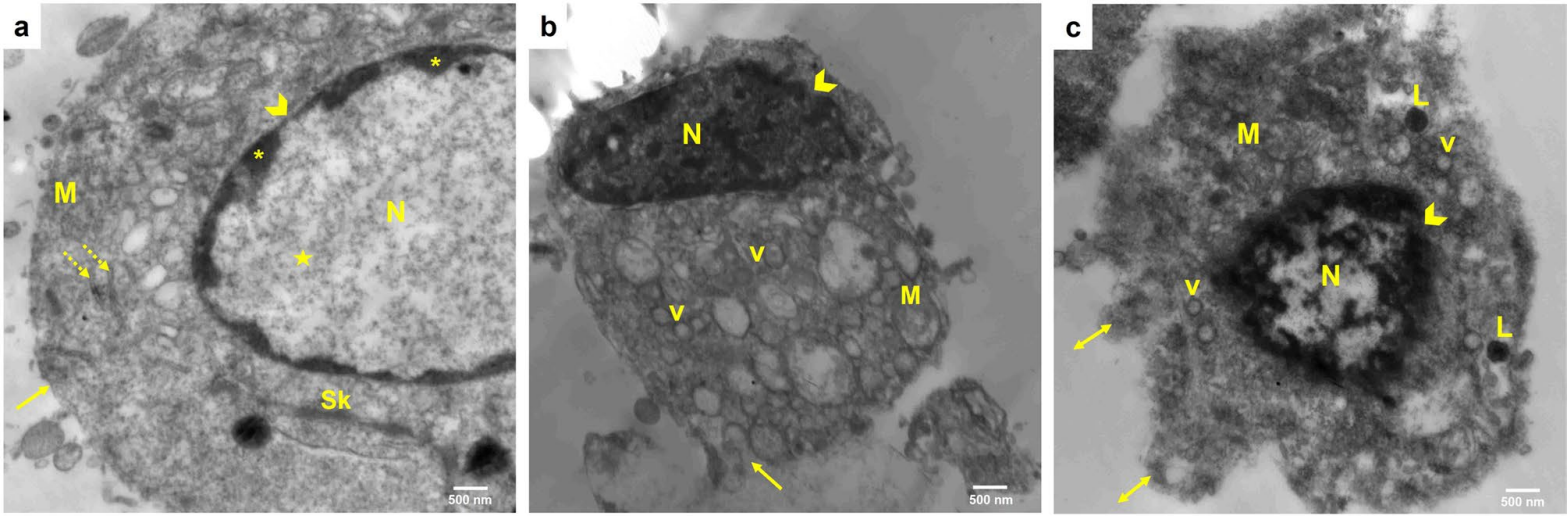
An electron micrograph of tongue carcinoma SCC154 cells showing (**a**) untreated control group (**b**) GLE group (**c**) GNPs group. Nucleus (N), euchromatin (star), heterochromatin (asterisks), nuclear membrane (arrowhead), cytoskeleton (Sk), mitochondria (M), rough endoplasmic reticulum (dotted arrows), plasma membrane (arrow), vacuoles (v), lysosomes (L), plasma membrane projections (double head arrows) (a-c; scale bar = 500 nm)

Ultrastructural analysis of the untreated group disclosed cells with large, amorphous nucleus, intact nuclear membrane, and a predominance of euchromatin alongside small heterochromatin aggregates. The cytoplasm contained endoplasmic reticulum, cytoskeleton, and mitochondria. Cells displayed smooth, undamaged plasma membrane (Fig. 4a). The graviola-treated group showed a shrunken and degenerated cell. The nucleus appeared in an abnormal shape and size, and with an irregular nuclear membrane. Numerous cytoplasmic vacuoles and enlarged mitochondria were found in the cytoplasm. The plasma membrane appeared discontinuous (Fig. 4b). The cells in the nano-graviola-treated group appeared shrunken and showed cellular blebbing. Nuclear changes involved an irregularly shaped nucleus with chromatin condensation and a disrupted nuclear membrane. The cytoplasm displayed enlarged mitochondria and lysosomes. Cytoplasmic vacuolization was detected. The plasma membrane was indistinct and fragmented (Fig. 4c).

## Discussion

Targeted cancer therapies provide great specificity in treatment; however, they often face challenges such as resistance mechanisms and difficulties in delivery. As a result, there is an ongoing need to develop new therapies to address these issues [17].

Graviola leaves have shown cytotoxic effects on a range of cancer cell lines, triggering apoptosis via different mechanisms, suggesting their potential as effective anticancer agents [18, 19]. Natural compounds delivered via nano‑formulations became popular in cancer therapy since these nano-formulations encapsulate bioactive substances, shielding them from degradation and increasing their body absorption [20]. The efficacy of nano-delivery systems was proven in overcoming drug resistance in colorectal, breast, and ovarian cancers [21–23]. These findings support our approach of utilizing nano-encapsulated graviola to enhance its bioavailability and selectively regulate key oncogenic pathways.

In the current work, GLE and GNPs exhibited significantly higher cytotoxic, apoptotic, and DNA-damaging effects, along with pronounced cell cycle arrest, compared to the untreated group, with significantly better results in the nano-formulation compared to its free counterpart. This might be attributed to the high entrapment efficiency of GNPs synthesized in this study, which enabled sustained drug release and improved compound stability, thereby enhancing the bioavailability of the active agents within cancer cells and contributing to the observed cytotoxic effects. These findings aligned with Magadi et al. [24], who reported the cytotoxic effects of graviola extract on SCC-25 cells using the MTT test and flow cytometry. Similarly, Mary et al. [25] demonstrated dose-dependent inhibition of SCC-15 cells following treatment with graviola extract. However, these studies utilized non-nano formulations, neglecting the enhanced efficacy of nano-encapsulation in increasing cytotoxicity and DNA damage.

Graviola extract likely induced apoptosis through the mitochondrial-mediated mechanism by breaking the mitochondrial membrane, resulting in G0/G1 phase cell cycle arrest [26, 27]. Additionally, this study utilized nano-encapsulation for GLE, a method known to enhance the cytotoxic effects of anticancer agents by increasing reactive oxygen species production, a key trigger of apoptosis in various types of cancer [28, 29].

**Fig. 5:**
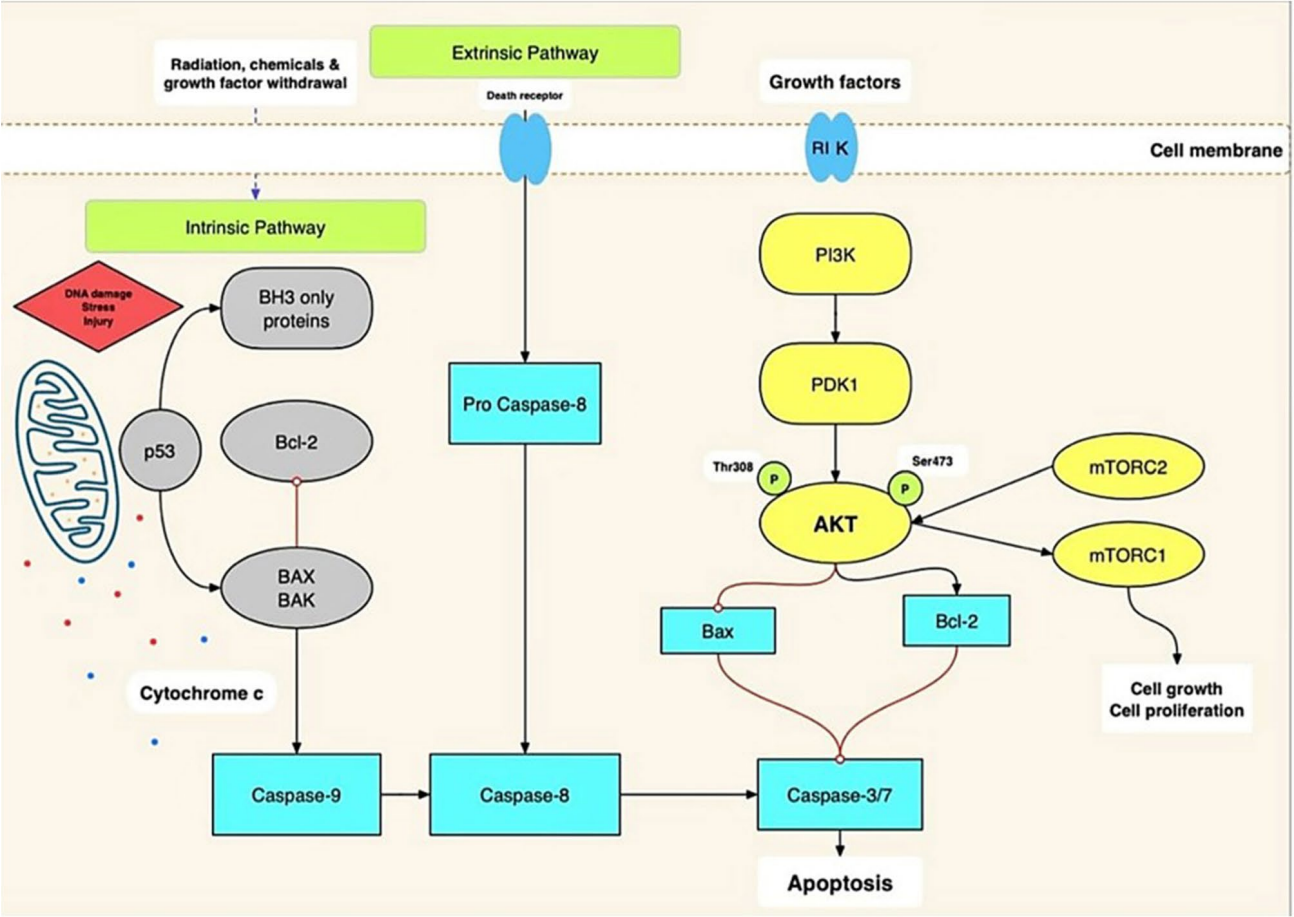
The intrinsic, extrinsic, and PI3K/AKT/mTOR signaling pathways involved in the regulation of apoptosis and cell proliferation. The red lines with rounded ends indicate inhibition, while black arrows indicate stimulation

A key aspect of this study is assessing the molecular expression of glycogen synthase kinase-3β (GSK-3β) and the PI3K/AKT/mTOR signaling pathway. Tumorigenesis is significantly influenced by the PI3K/AKT/mTOR pathway’s activation. This pathway contributes to resistance against anticancer therapies. Activated AKT phosphorylates and inactivates pro-apoptotic markers such as BAD and caspase-9, allowing anti-apoptotic proteins like Bcl-2 and Bcl-xL to preserve mitochondrial integrity and prevent cell death. In contrast, inhibition of this pathway shifts the balance towards apoptosis by promoting cytochrome c release and activating caspase-9 and caspase-3. These events clarify how PI3K/AKT/mTOR signaling functions not just upstream, but also as a master regulator of downstream apoptotic control [30, 31] (Fig.5). Accordingly, small-molecule inhibitors targeting PI3K have shown promising results in impeding tumor progression [32, 33].

GSK-3β is a serine/threonine kinase that possess multiple roles in cellular processes and signaling pathways. Researchers have explored the inhibition of GSK-3β as a potential treatment for cancer. Inhibiting GSK-3β has been associated with a reduction in cancer cell growth across several types of malignancies, including prostate, and colon cancers and leukemia [34–36].

The current work revealed a significant downregulation of PI3K, AKT, mTOR, and GSK-3β mRNA levels in cancer cells treated with GLE and GNPs compared to the untreated group, with a significant downregulation in GNPs compared to its free counterpart. These molecular alterations aligned with the observed cytotoxic and pro-apoptotic effects, suggesting their involvement in the treatment response. Consistent with our findings, Shorning et al. [37] emphasized the critical role of PI3K/AKT/mTOR in prostate cancer progression and treatment resistance. Similarly, Kubczak et al. [38] demonstrated graviola’s suppression of several signaling pathways, inhibiting metastasis and tumorigenicity of cancer cells.

In the present investigation, the ultrastructural analysis demonstrated significant degenerative alterations in tongue carcinoma SCC154 cells following treatment with both GLE and GNPs, with these alterations being aggravated in the nano-encapsulated group. These findings support the cytotoxic and apoptosis-inducing effects observed in earlier assays.

In the current work, the anticancer effect of graviola and its nano formulation could be attributed to the phenolic compounds identified in GLE. Oleuropein was identified as the major compound in our GLE. Oleuropein has been shown to suppress cancer cell proliferation and enhance apoptosis by targeting the AKT signaling pathway. It increases the pro-apoptotic protein Bax and decreases the anti-apoptotic protein Bcl-2 [39]. Oleuropein also exhibited strong binding affinity to key components of the PI3K/AKT/mTOR pathway, indicating its potential to inhibit this cascade [40]. Additionally, oleuropein has been reported to induce cell death in thyroid and cervical cancer cells [41, 42]. Catechin, identified as the second major compound in our extract, has also been proved to trigger apoptosis in cervical cancer cells [43, 44]. It inhibits the PI3K/AKT signaling pathway in gastric cancer cells by reducing phosphorylated AKT levels and promoting apoptosis in a dose-dependent manner [45]. Moreover, pyrogallol boosted the efficacy of the chemotherapeutic agent cisplatin and induced apoptosis in ovarian cancer cells [46]. Other bioactive compounds identified in our extract, such as gallic acid and ellagic acid, exerted anticancer effects by promoting apoptosis and cell cycle arrest via affecting the PI3K/AKT signaling pathway [47, 48]. Caffeic acid acts as an antioxidant in normal cells but as a prooxidant in cancer cells. It has been shown to increase apoptotic changes in the HeLa and ME-180 cell lines [49].

### Limitations

This study has some limitations. First, gene expression was evaluated only at the mRNA level using qPCR, without assessing protein levels via western blotting or ELISA. While mRNA analysis provides valuable insight into gene regulation, it does not necessarily reflect actual protein expression; therefore, future studies incorporating proteomic analysis are encouraged. Second, the absence of separate vehicle control groups for the solvent and carrier used in the treatment formulations limits the ability to attribute observed effects solely to the active agents. Although an untreated control group was included, vehicle controls would have strengthened data interpretation. Lastly, the exclusive use of in vitro models, while useful for controlled cellular analysis, may not fully reflect the complexity of in vivo biological systems, thereby limiting the translational value of the findings. Therefore, planned animal studies are suggested to further assess the therapeutic efficacy, safety, and physiological relevance of nano-encapsulated graviola in a more complex biological context. 

## Conclusion

This study is the first to demonstrate that nano-encapsulated graviola exhibits promising anticancer activity against tongue carcinoma cells by inducing cytotoxicity, cell cycle arrest, and apoptosis. These effects were partially mediated through inhibition of the PI3K/AKT/mTOR signaling pathway. Nano-formulation enhanced graviola’s bioavailability, stability, and targeted delivery, further improving its therapeutic efficacy. While these findings support its potential as a complementary agent in oral squamous cell carcinoma and possibly other cancers, further in vivo studies and clinical trials are essential to confirm its safety and therapeutic value. Therefore, nano-graviola cannot be yet recommended as a standalone or alternative treatment.

## Supplementary Information

Supplementary Material 1.

## Data Availability

All data generated or analyzed during this study are included in this published article.

## Abbreviations

AKT: Protein kinase B
EDC: 1-Ethyl-3-(3-dimethylaminopropyl)-N’-ethyl-carbodiimide
EE: Entrapment efficiency
GLE: Graviola leaves extract
GNPs: Graviola nanoparticles
GSK-3β: Glycogen synthase kinase-3 beta
HPLC: High-performance liquid chromatography
mTOR: Mammalian target of rapamycin
NPs: Nanoparticles
NHS: N-hydroxysuccinimide
PBS: Phosphate-buffered saline
PDI: Polydispersity index
PEG-NH2: Polyethylene glycol amine
PI: Propidium iodide
PI3K: Phosphatidylinositol-3-kinase
PLGA: Poly (D, L-lactide-co-glycolide)
