# Supplementary figures and images for: Revealing the anticancer potential of nano-encapsulated graviola extract on tongue carcinoma (SCC154) cell line: targeting the PI3K/AKT/mTOR pathway (in vitro study)
Source: BMC Complement Med Ther. 2025 Oct 2;25:352. doi: 10.1186/s12906-025-05113-4 (PMC12490109; doi:10.1186/s12906-025-05113-4)

## Slide 1
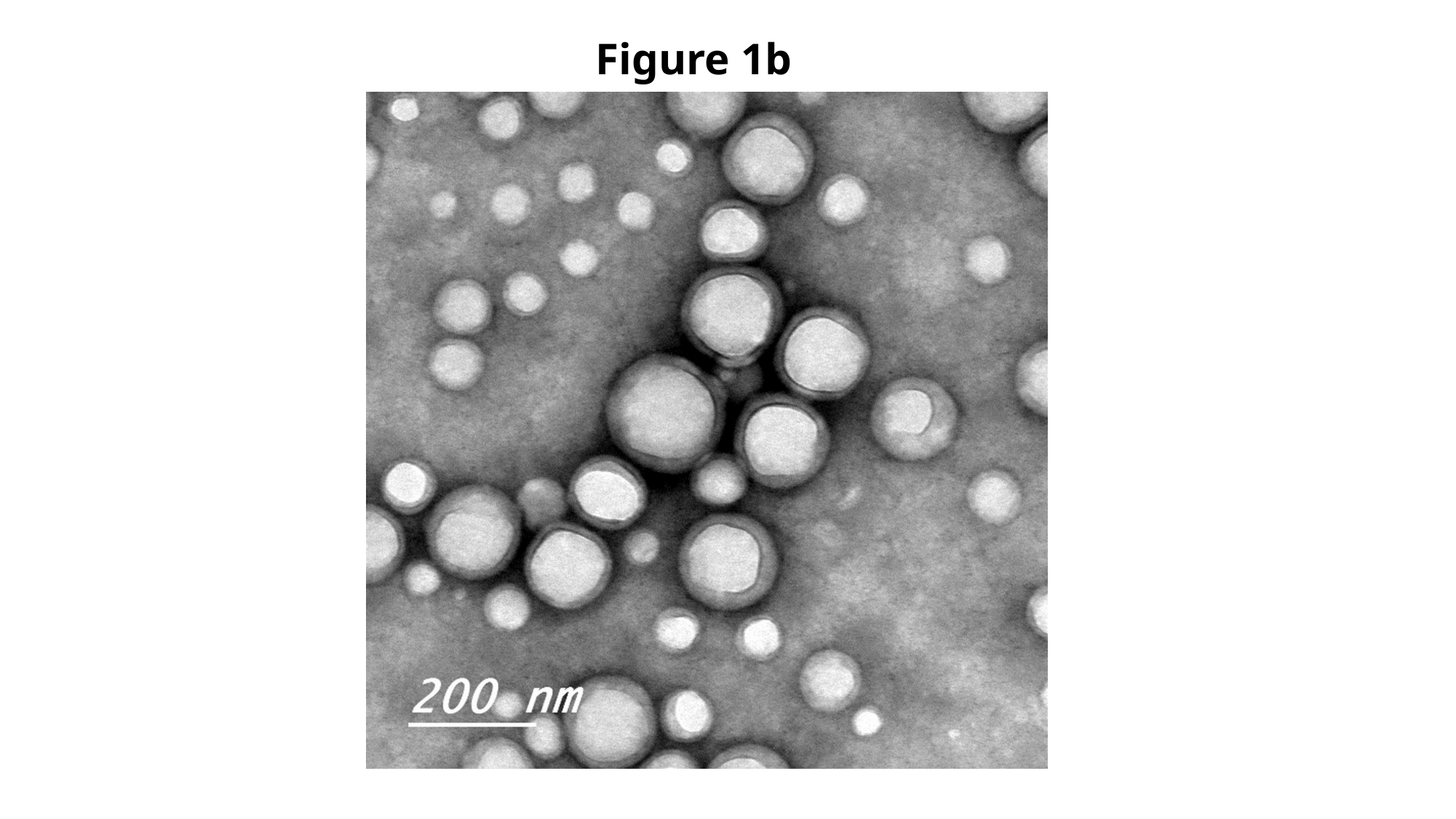

Figure 1b

## Slide 2
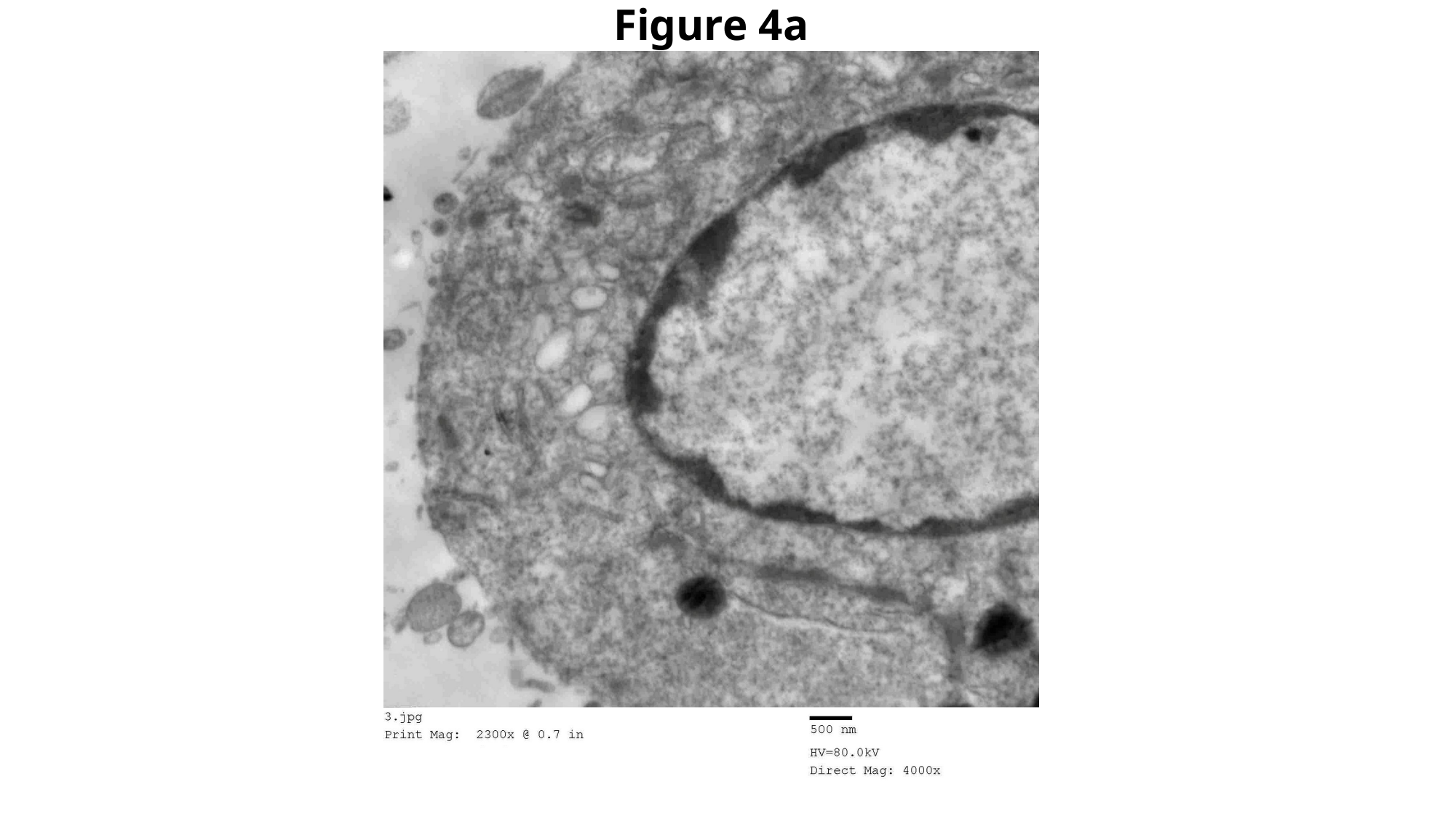

Figure 4a

## Slide 3
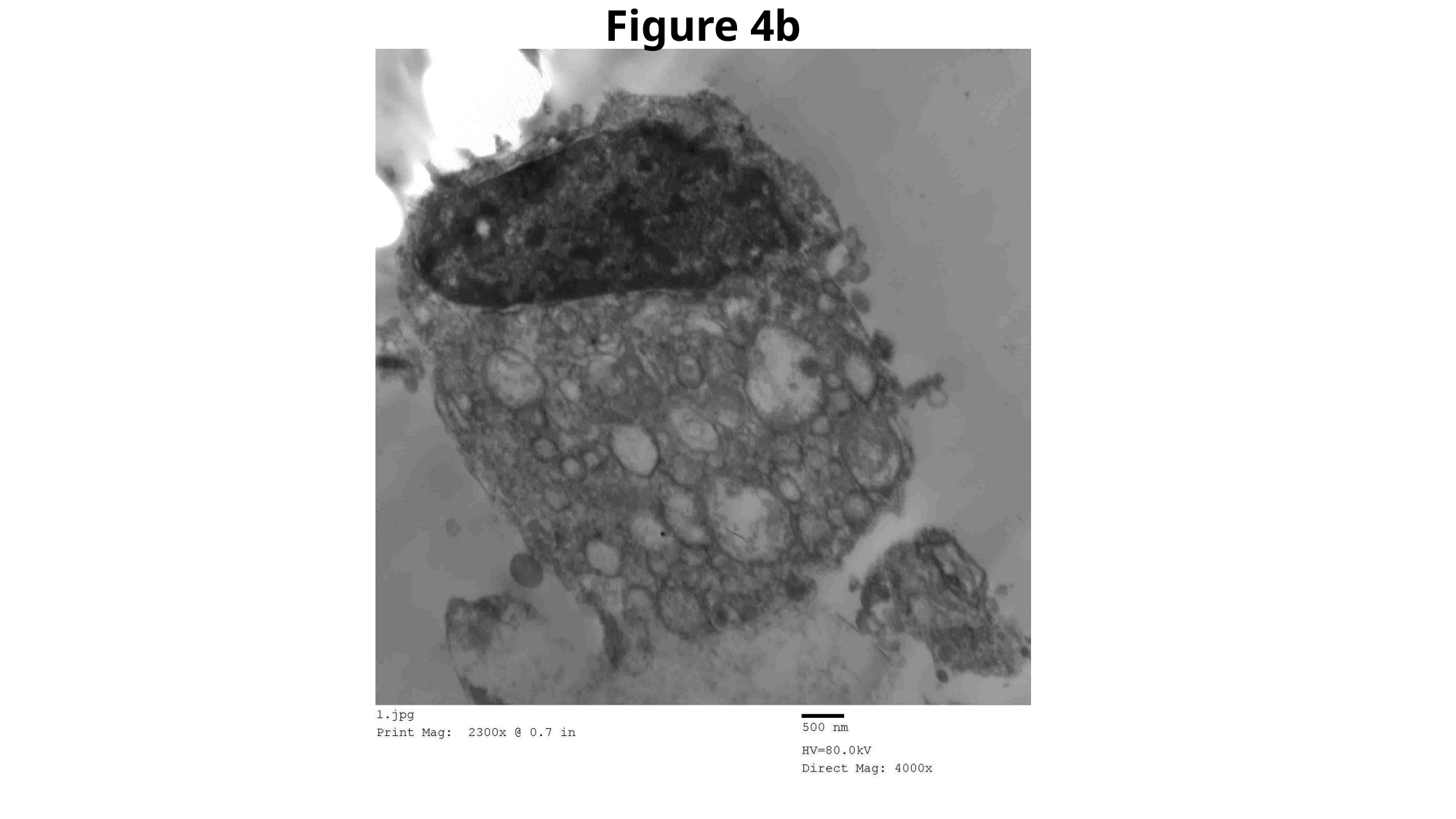

Figure 4b

## Slide 4
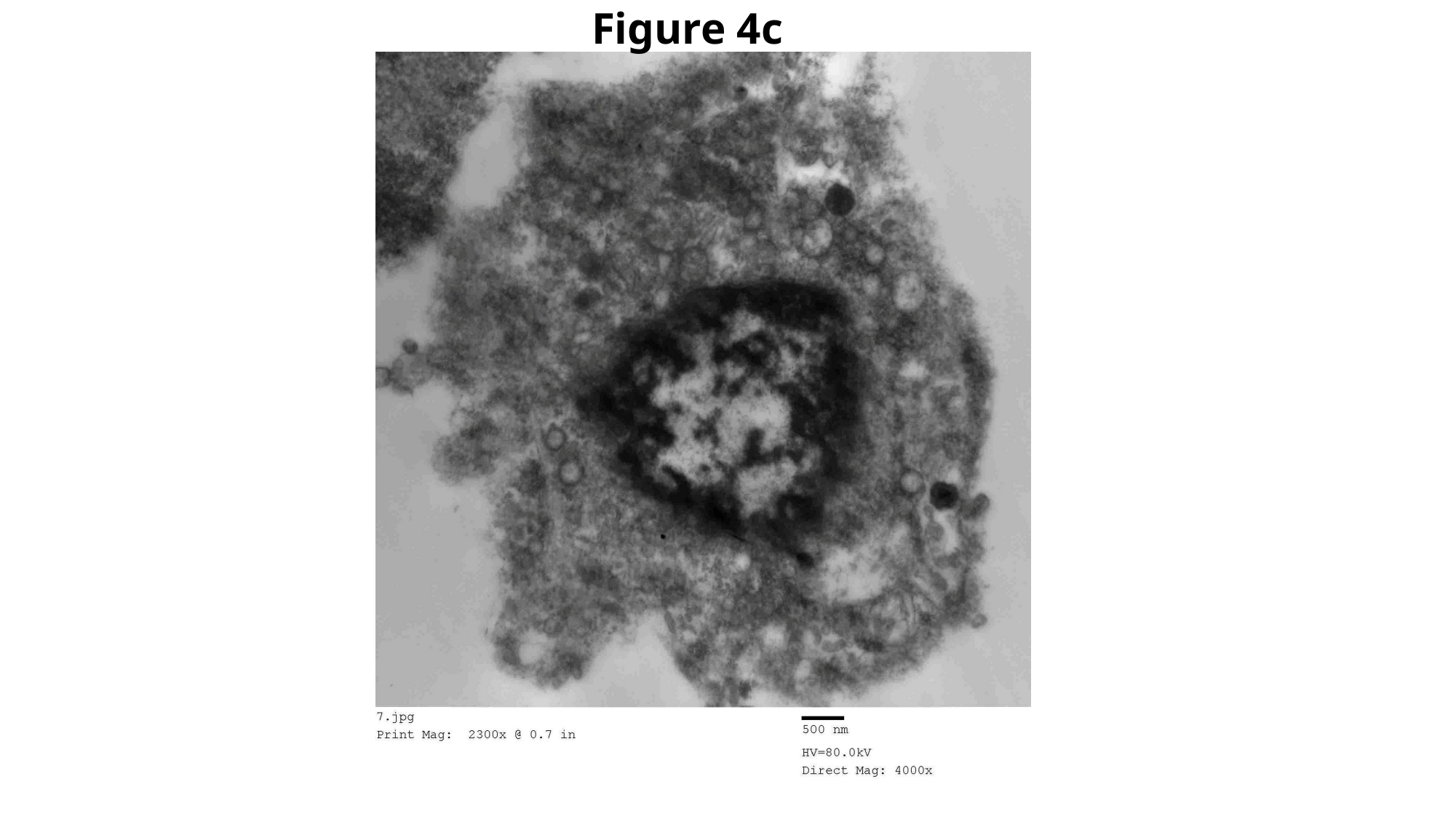

Figure 4c

Supplement: Supplementary file 1 — Supplementary Material 1. [file 12906_2025_5113_MOESM1_ESM.pptx]
